# Supplementary material for: Analysis on the impact of dynamic innovation investment strategy of green supply chain enabled by blockchain
Source: PeerJ Comput Sci. 2024 Apr 29;10:e2002. doi: 10.7717/peerj-cs.2002 (PMC11065413; doi:10.7717/peerj-cs.2002)
Supplement: Supplemental Information 1 [file peerj-cs-10-2002-s001.docx]

**PROOF PROCESS**

**Proof of Proposition 1**

For the sake of notational simplicity, the following proof procedure is omitted t. Using the inverse induction method, we first solve the strategy in the post-innovation environment. According to the theory of continuum dynamic programming, the optimal value function $W_{F}^{C}(G)$ of the post-innovation green supplier and the optimal value function $W_{MN}^{C}$ of the cooperating Internet Platforms satisfy the following Hamilton-Jacobi-Bellman (HJB) equations for all $G\geq0$ under the case of horizontal cooperation among franchisors

(1)

(2)

where , are the first order derivatives of the optimal value functions $W_{F}^{C}$, $W_{MN}^{C}$ about$G$, respectively. From the first-order optimality condition, it follows that.

Substituting the above equations into Eqs. (1)(2) gives the HJB equation

(3)

(4)

Based on the structure of Eqs. (3)(4), it is hypothesized that$W_{F}^{C}=d_{11}G+d_{01},W_{MN}^{C}=d_{22}G+d_{02}$, where $d_{11}>0,d_{01}>0,d_{22}>0,d_{02}>0$ is the coefficient to be determined. Substituting $W_{F}^{C}\text{、}W_{MN}^{C}$ and first-order derivatives into Eqs. (3)(4), each of the coefficients to be determined can be obtained according to the constant relationship. Substituting back the corresponding equations can obtain the strategies and value functions after innovation in proposition 1.

The HJB equation for the pre-innovation green supplier and collaborative internet platform are

(5)

(6)

where $V_{F}^{C}\text{、}V_{MN}^{C}$ are the optimal value functions of the green supplier and collaborative internet platform, respectively, for the entire program period, and from the first-order optimality condition we get

.

Substituting the above equations into Eqs. (5)(6) yields the HJB equation

Based on the structure of the above equation, it is hypothesized that $V_{F}^{C}=d_{4}G+d_{04},V_{MN}^{C}=d_{3}G+d_{03}$, where $d_{4}>0,d_{04}>0,d_{3}>0,d_{03}>0$ are the coefficients to be determined. Substituting $V_{F}^{C}\text{、}V_{MN}^{C}$ and the first-order derivative, each coefficient to be determined can be obtained according to the constant relationship. Substituting the corresponding equations, we can get the strategies before innovation in proposition 1 and the value function in the whole planning period. Substituting each optimal strategy into the state evolution equation and solving it, the time evolution path of franchise goodwill can be obtained. Proposition 1 is proved.

**Proof of Proposition 2**

The proof procedure is similar to Proposition 1, with the difference that the HJB equations for the green supplier ($F$), and the two Internet platforms ($M\text{、}N$) before and after the innovation need to be written separately. In order to save space, it is not repeated here.

**Proof of Proposition 3**

The proof process is similar to Proposition 1, except that the HJB equations for the green supplier ($F$) and the cooperative Internet platform ($M$), and the Internet platform $N$need to be written. In order to save space, we will not repeat it here.

**Proof of Proposition 4**

From Propositions 1-3, we have

$a_{1}^{C}-a_{1}^{B}\text{=}\frac{\theta_{1}d_{4}}{k_{F}}-\frac{\theta_{1}d_{8}}{k_{F}}=\frac{\theta_{1}(d_{4}-d_{8})}{k_{F}}$;$d_{4}-d_{8}=\frac{2\rho_{F1}\omega_{1}\text{+}\chi d_{11}(1+\varphi)}{r\text{+}\chi+\delta_{1}}-\frac{2\omega_{1}\rho_{F1}\text{+}\chi d_{5}(1+\varphi)}{r+\chi+\delta_{1}}=\frac{\chi d_{11}(1+\varphi)-\chi d_{5}(1+\varphi)}{r\text{+}\chi+\delta_{1}}$;

$d_{11}-d_{5}=\frac{2\omega_{2}\rho_{F2}}{r+\delta_{2}}-\frac{2\omega_{2}\rho_{F2}}{r+\delta_{2}}=0$;$a_{1}^{H}-a_{1}^{C}=\frac{\theta_{1}(d_{14}-d_{4})}{k_{F}}$;

$d_{14}-d_{4}=\frac{(2\rho_{F1}+\rho_{M1})\omega_{1}+\chi d_{12}(1+\varphi)}{r+\chi+\delta_{1}}-\frac{2\rho_{F1}\omega_{1}\text{+}\chi d_{11}(1+\varphi)}{r\text{+}\chi+\delta_{1}}=\frac{\rho_{M1}\omega_{1}+\chi(1+\varphi)(d_{12}-d_{11})}{r\text{+}\chi+\delta_{1}}$;

$d_{12}-d_{11}=\frac{(2\rho_{F2}+\rho_{M2})\omega_{2}}{r+\delta_{2}}-\frac{2\rho_{F2}\omega_{2}}{r+\delta_{2}}=\frac{\rho_{M2}\omega_{2}}{r+\delta_{2}}>0$;

$\therefore a_{1}^{H}>a_{1}^{C}=a_{1}^{B}$.

as can be seen from the same principle $a_{2}^{H}>a_{2}^{C}=a_{2}^{B}$;$u_{M1}^{H}>u_{M1}^{C}>u_{M1}^{B}$;$u_{M2}^{H}>u_{M2}^{C}>u_{M2}^{B}$;$u_{N1}^{C}>u_{N1}^{B}=u_{N1}^{H}$;$u_{N2}^{C}>u_{N2}^{B}=u_{N2}^{H}$.

**Proof of Proposition 5**

From Propositions 1-3, we have

$s_{x1}^{C}-s_{x1}^{B}=\frac{\rho_{x1}\eta_{1}-\rho_{y1}\xi_{1}+\alpha_{1}d_{3}}{k_{xs}}-\frac{\rho_{x1}\eta_{1}+\alpha_{1}d_{9}}{k_{xs}}=\frac{\alpha_{1}(d_{3}-d_{9})-\rho_{y1}\xi_{1}}{k_{xs}}$；$d_{3}-d_{9}=\frac{\rho_{N1}\omega_{1}+\chi(1+\varphi)(d_{22}-d_{6})}{r\text{+}\chi+\delta_{1}}$;

$d_{22}-d_{6}=\frac{\rho_{N2}\omega_{2}}{r+\delta_{2}}$；即$s_{x1}^{C}-s_{x1}^{B}=\frac{\alpha_{1}\rho_{N2}\omega_{2}\chi(1+\varphi)+\alpha_{1}\rho_{N1}\omega_{1}(r+\delta_{2})-\rho_{N1}\xi_{1}(r\text{+}\chi+\delta_{1})(r+\delta_{2})}{k_{xs}(r\text{+}\chi+\delta_{1})(r+\delta_{2})}$;

$\alpha_{1}\omega_{2}\chi(1+\varphi)+\alpha_{1}\omega_{1}(r+\delta_{2})-\xi_{1}(r\text{+}\chi+\delta_{1})(r+\delta_{2})>0$,$s_{x1}^{C}-s_{x1}^{B}>0$;

$s_{M1}^{H}-s_{M1}^{C}=\frac{\rho_{F1}\left(\eta_{1}-\xi_{1}\right)+\rho_{M1}\eta_{1}+\alpha_{1}d_{14}}{k_{Ms}}-\frac{\rho_{M1}\eta_{1}-\rho_{M1}\xi_{1}+\alpha_{1}d_{3}}{k_{Ms}}=\frac{\rho_{F1}\eta_{1}-\rho_{F1}\xi_{1}+\rho_{M1}\xi_{1}+\alpha_{1}d_{14}-\alpha_{1}d_{3}}{k_{Ms}}$;

$d_{14}-d_{3}=\frac{(2\rho_{F1}+\rho_{M1})\omega_{1}+\chi d_{12}(1+\varphi)}{r+\chi+\delta_{1}}-\frac{\rho_{M1}\omega_{1}+\rho_{N1}\omega_{1}+\chi d_{22}(1+\varphi)}{r\text{+}\chi+\delta_{1}}=\frac{2\rho_{F1}\omega_{1}-\rho_{N1}\omega_{1}+\chi d_{12}(1+\varphi)-\chi d_{22}(1+\varphi)}{r+\chi+\delta_{1}}$;

$d_{12}-d_{22}=\frac{(2\rho_{F2}+\rho_{M2})\omega_{2}}{r+\delta_{2}}-\frac{\left(\rho_{M2}+\rho_{N2}\right)\omega_{2}}{r+\delta_{2}}=\frac{2\rho_{F2}\omega_{2}-\rho_{N2}\omega_{2}}{r+\delta_{2}}>0$;

$$∵\eta_{1}>\xi_{1}$$

$\therefore s_{M1}^{H}>s_{M1}^{C}>s_{M1}^{B}$.

as can be seen from the same principle $\alpha_{2}\omega_{2}-\xi_{2}(r+\delta_{2})>0$,$s_{M2}^{H}>s_{M2}^{C}>s_{M2}^{B}$;$s_{N1}^{C}>s_{N1}^{B}=s_{N1}^{H}$;$s_{N2}^{C}>s_{N2}^{B}=s_{N2}^{H}$.

**Proof of Proposition 6**

From Propositions 1-3, we have

$G_{1\infty}^{C}-G_{1\infty}^{B}=\frac{1}{\delta_{1}}\left[\frac{\theta_{1}^{2}(d_{4}-d_{8})}{k_{F}}\text{+}\frac{\alpha_{1}(\alpha_{1}d_{3}-\alpha_{1}d_{9}-\rho_{N1}\xi_{1})}{k_{Ms}}+\frac{\alpha_{1}(\alpha_{1}d_{3}-\alpha_{1}d_{10}-\rho_{M1}\xi_{1})}{k_{Ns}}\right]$;$d_{4}-d_{8}=\frac{\chi(1+\varphi)(d_{11}-d_{5})}{r\text{+}\chi+\delta_{1}}$;

$d_{11}-d_{5}=\frac{2\rho_{F2}\omega_{2}}{r+\delta_{2}}-\frac{2\omega_{2}\rho_{F2}}{r+\delta_{2}}=0$.

$\alpha_{1}\omega_{2}\chi(1+\varphi)+\alpha_{1}\omega_{1}(r+\delta_{2})-\xi_{1}(r\text{+}\chi+\delta_{1})(r+\delta_{2})>0$,$\alpha_{1}(\alpha_{1}d_{3}-\alpha_{1}d_{9}-\rho_{N1}\xi_{1})>0$;

$d_{3}-d_{10}=\frac{\rho_{M1}\omega_{1}+\rho_{N1}\omega_{1}+\chi d_{22}(1+\varphi)}{r\text{+}\chi+\delta_{1}}-\frac{\rho_{N1}\omega_{1}+\chi d_{7}(1+\varphi)}{r+\chi+\delta_{1}}=\frac{\rho_{M1}\omega_{1}+\chi(1+\varphi)(d_{22}-d_{7})}{r+\chi+\delta_{1}}$;

$d_{22}-d_{7}=\frac{\left(\rho_{M2}+\rho_{N2}\right)\omega_{2}}{r+\delta_{2}}-\frac{\rho_{N2}\omega_{2}}{r+\delta_{2}}=\frac{\rho_{M2}\omega_{2}}{r+\delta_{2}}>0$.

$\therefore G_{1\infty}^{H}>G_{1\infty}^{C}>G_{1\infty}^{B}$;$G_{2\infty}^{H}>G_{2\infty}^{C}>G_{2\infty}^{B}$。

**Proof of Proposition 7**

From Propositions 1-6, we have

$$\begin{matrix} V_{MN}-V_{M}-V_{N}=\left(d_{3}G^{M+N}-d_{9}G^{B}-d_{10}G^{B}\right)+\left(d_{03}-d_{010}-d_{09}\right)=\mathop{d_{3}\left(G^{M+N}-G^{B}\right)}\limits_{>0} +\left(d_{03}-d_{09}-d_{010}\right) \\ d_{03}-d_{010}-d_{09}=\left\{\frac{\left[\rho_{M1}\eta_{1}-\xi_{1}\rho_{N1}+\alpha_{1}d_{3}\right]^{2}}{2\left(r\text{+}\chi\right)k_{Ms}}-\frac{\left[\rho_{M1}\eta_{1}+\alpha_{1}d_{9}\right]^{2}}{2\left(r+\chi\right)k_{Ms}}-\frac{2\left(d_{10}\alpha_{1}-\rho_{N1}\xi_{1}\right)\left(\rho_{M1}\eta_{1}+\alpha_{1}d_{9}\right)}{2\left(r+\chi\right)k_{Ms}}\right\} \\ +\left\{\frac{\left[\eta_{1}\rho_{N1}-\xi_{1}\rho_{M1}+\alpha_{1}d_{3}\right]^{2}}{2\left(r\text{+}\chi\right)k_{Ns}}-\frac{\left[\eta_{1}\rho_{N1}+\alpha_{1}d_{10}\right]^{2}}{2\left(r+\chi\right)k_{Ns}}-\frac{\left[\eta_{1}\rho_{N1}+\alpha_{1}d_{10}\right]^{2}}{\left(r+\chi\right)k_{Ns}}\right\}+\left\{\frac{\left[\rho_{M1}\lambda_{1}+\rho_{N1}\gamma_{1}\right]^{2}}{2\left(r\text{+}\chi\right)k_{Mu}}-\frac{\rho_{M1}\rho_{M1}\lambda_{1}\lambda_{1}}{2\left(r+\chi\right)k_{Mu}}-\frac{\rho_{N1}\gamma_{1}\rho_{M1}\lambda_{1}}{\left(r+\chi\right)k_{Mu}}\right\} \\ +\left\{\frac{\left[\rho_{M1}\gamma_{1}+\rho_{N1}\lambda_{1}\right]^{2}}{2\left(r\text{+}\chi\right)k_{Nu}}-\frac{\rho_{M1}\gamma_{1}\lambda_{1}\rho_{N1}}{\left(r+\chi\right)k_{Nu}}-\frac{\lambda_{1}\lambda_{1}\rho_{N1}\rho_{N1}}{2\left(r+\chi\right)k_{Nu}}\right\}+\left\{\frac{d_{3}\theta_{1}\theta_{1}d_{4}}{\left(r\text{+}\chi\right)k_{F}}-\frac{d_{9}\theta_{1}\theta_{1}d_{8}}{\left(r+\chi\right)k_{F}}-\frac{d_{10}\theta_{1}\theta_{1}d_{8}}{\left(r+\chi\right)k_{F}}\right\}+\left\{\frac{\chi d_{02}}{\left(r\text{+}\chi\right)}-\frac{\chi d_{06}}{\left(r+\chi\right)}-\frac{\chi d_{07}}{\left(r+\chi\right)}\right\} \end{matrix}$$

$d_{03}-d_{010}-d_{09}=\frac{\left(\alpha_{1}d_{10}-\xi_{1}\rho_{N1}\right)^{2}}{2\left(r\text{+}\chi\right)k_{MS}}+\frac{\left(\alpha_{1}d_{9}-\xi_{1}\rho_{M1}\right)^{2}}{2\left(r\text{+}\chi\right)k_{Ns}}+\frac{\left(\rho_{N1}\gamma_{1}\right)^{2}}{2\left(r\text{+}\chi\right)k_{Mu}}+\frac{\left(\rho_{M1}\gamma_{1}\right)^{2}}{2\left(r\text{+}\chi\right)k_{Nu}}+\frac{\chi}{\left(r\text{+}\chi\right)}\left[d_{01}-d_{06}-d_{07}\right]>0$；

$$\begin{matrix} d_{02}-d_{06}-d_{07}=\frac{\left[\rho_{M2}\eta_{2}-\rho_{N2}\xi_{2}+d_{1}\alpha_{2}\right]^{2}}{2rk_{Ms}}+\frac{\left[\rho_{M2}\lambda_{2}+\rho_{N2}\gamma_{2}\right]^{2}}{2rk_{Mu}}+\frac{\left[\rho_{N2}\eta_{2}-\rho_{M2}\xi_{2}+d_{1}\alpha_{2}\right]^{2}}{2rk_{Ns}}+\frac{\left[\rho_{M2}\gamma_{2}+\rho_{N2}\lambda_{2}\right]^{2}}{2rk_{Nu}}+\frac{d_{2}d_{1}\theta_{2}\theta_{2}}{rk_{F}} \\ -\frac{\left[\rho_{M2}\eta_{2}+\alpha_{2}d_{6}\right]^{2}}{2rk_{Ms}}-\frac{\left(d_{6}\alpha_{2}-\rho_{M2}\xi_{2}\right)\left(\rho_{N2}\eta_{2}+\alpha_{2}d_{7}\right)}{rk_{Ns}}-\frac{\rho_{M2}\rho_{M2}\lambda_{2}\lambda_{2}}{2rk_{Mu}}-\frac{\rho_{N2}\rho_{M2}\gamma_{2}\lambda_{2}}{rk_{Nu}}-\frac{d_{6}\theta_{2}\theta_{2}d_{5}}{rk_{F}} \\ -\frac{\left[\rho_{N2}\eta_{2}+\alpha_{2}d_{7}\right]^{2}}{2rk_{Ns}}-\frac{\left(d_{7}\alpha_{2}-\rho_{N2}\xi_{2}\right)\left(\rho_{M2}\eta_{2}+\alpha_{2}d_{6}\right)}{rk_{Ms}}-\frac{\rho_{N2}\rho_{N2}\lambda_{2}\lambda_{2}}{2rk_{Nu}}-\frac{\rho_{N2}\gamma_{2}\rho_{M2}\lambda_{2}}{rk_{Mu}}-\frac{d_{7}\theta_{2}\theta_{2}d_{5}}{rk_{F}} \end{matrix}$$

$d_{02}-d_{06}-d_{07}=\frac{\left[\alpha_{2}d_{7}-\rho_{N2}\xi_{2}\right]^{2}}{2rk_{Ms}}+\frac{\left(\rho_{N2}\gamma_{2}\right)^{2}}{2rk_{Mu}}+\frac{\left[d_{6}\alpha_{2}-\rho_{M2}\xi_{2}\right]^{2}}{2rk_{Ns}}+\frac{\left(\rho_{M2}\gamma_{2}\right)^{2}}{2rk_{Nu}}>0$；$\therefore V_{MN}>V_{M}+V_{N}$。

as can be seen from the same principle $V_{FM}>V_{F}+V_{M}$、$V^{H}>V^{C}>V^{B}$.
